# Neoadjuvant intra-arterial versus intravenous chemotherapy in colorectal cancer

**DOI:** 10.1097/MD.0000000000028312

**Published:** 2021-12-23

**Authors:** Shu hui Peng, Hussein Said Mbarak, Yan-Hui Li, Cong Ma, Quan-Liang Shang, Zhu Chen, Du-Jun Bian, En-Hua Xiao

**Affiliations:** Department of Radiology, The Second Xiangya Hospital of Central South University, Changsha, Hunan Province, China.

**Keywords:** colorectal cancer, intravenous chemotherapy, neoadjuvant chemotherapy, regional arterial infusion chemotherapy

## Abstract

To investigate the clinical benefits of transcatheter arterial infusion chemotherapy compared with intravenous chemotherapy in patients with colorectal cancer (CRC).

From May 2013 to March 2018, 83 patients (50 men and 33 women) with surgically proven CRC were retrospectively included. Before surgery, 62 patients received conventional systemic chemotherapy, and 21 transcatheter arterial chemotherapy. Basic characteristics, disease control rate (DC), adverse reactions, postoperative complications, and toxicity profiles were collected and compared between the 2 groups.

The sigmoid colon (43.37%) was the most common primary tumor location, and the least was the transverse colon (6.02%). Most lesions invaded the subserosa or other structures T3-4 (78.31%), and other lesions invaded the muscular layer T1-2 (21. 69%). The overall DC was 80.65% in the intravenous chemotherapy group and 90.48% in the arterial chemotherapy group (*P* < .05). Adverse events included myelosuppression and gastrointestinal reactions such as nausea, vomiting, diarrhea, abnormal liver function, and neurotoxicity, which were significantly less common in the intra-arterial group than in the intravenous group (*P* < .05). Postoperative complications included abdominal infection (11.29% vs 14.29%), intestinal obstruction (6.45% vs 4.76%), anastomotic bleeding (1.61% vs 0.00%), and anastomotic fistula (6.45% vs 4.76%) in the intravenous and intra-arterial groups, respectively (*P* > .05).

Preoperative transcatheter arterial infusion chemotherapy is a safe and effective neoadjuvant chemotherapy measure for CRC with fewer adverse reactions and a higher overall DC.

## 1 Introduction

Colorectal cancer (CRC) is the third most commonly diagnosed malignancy and the fourth leading cause of cancer-related death worldwide.^[1]^ In China, cancer incidence and cancer mortality rates for CRC increased from 2000 to 2011, and CRC was estimated to be the fifth leading cause of cancer-related death in 2015.^[2]^ Up to 50% of patients with CRC miss the opportunity of surgery at the time of diagnosis or will develop future metastases or local recurrence.^[3]^ For inoperable patients, resectability is improved with neoadjuvant chemotherapy.^[4]^ To this end, systemic chemotherapy remains the treatment mainstay. Downsizing tumor systemic chemotherapy is effective, but it usually causes higher systemic toxicity and adverse effects. Recent advances in image-guided interventions have improved the safety of intra-arterial catheterization techniques for managing malignancies.^[5]^ Regional arterial infusion chemotherapy (RAIC) increases the chemotherapeutic agent concentration around the tumor, thereby improving therapeutic responses and minimizing the drugs’ adverse effects. Hepatic metastases are considered a prognostic factor in CRC.^[6]^ To improve resectability or downsize tumors, CRCs with multiple liver metastases should be treated with neoadjuvant chemotherapy.^[4]^ However, responses to systemic chemotherapy are variable and even less effective than those of hepatic arterial infusion chemotherapy.^[7]^

This study aimed to report the utility, response, and assess the benefits of RAIC over conventional systemic chemotherapy in the treatment of CRC.

## 2 Patients and methods

### 2.1 Ethical considerations

This retrospective study was approved by the Hospital Medical Ethical Committee, which waived the requirement for patient informed consent.

### 2.2 Patients and data collection

We retrospectively reviewed patients aged <75 years with histologically confirmed CRC between May 2013 and March 2018 in the current study. Patients received neoadjuvant systemic chemotherapy or transcatheter arterial chemotherapy before surgery. The inclusion criteria were as follows: all patients diagnosed with CRC and confirmed by pathology results treated at our center from May 2013 to March 2018 and all patients who underwent neoadjuvant systemic chemotherapy or transcatheter arterial chemotherapy combined with surgical resection for CRC. The exclusion criteria were as follows: patients with a history of radiotherapy or biological therapy; patients with hepatic metastases or other distant metastases; patients who received postoperative adjuvant therapy other than chemotherapy, such as radiotherapy or biological therapy; and Karnofsky Performance Scale scores <70 points. Age, sex, preoperative symptoms, time of last follow-up, time of tumor recurrence and death, other coexisting malignancy or tumor-prone conditions, and histological reports were collected.

### 2.3 Treatment

Intravenous chemotherapy was administered using a regimen containing raltitrexed and oxaliplatin. A 15-minute intravenous raltitrexed (2.5 mg/m^2^) administration and a 180-minute oxaliplatin (100 mg/m^2^) infusion on the first day every 3 weeks. Intra-arterial chemotherapy was performed using femoral artery intubation according to the Seldinger technique. Arteriography of the superior or inferior mesenteric artery was performed to localize the tumor and identify the feeding arteries that were then selected or super-selected before chemo-infusion. Oxaliplatin (100–200 mg) was infused into the tumor feeding artery, followed by raltitrexed (2–4 mg) infusion until flow stasis in tumor vascularity was achieved. Intra-arterial chemo-infusion was performed at monthly intervals and repeated for up to 3 cycles if no disease progression or intolerable toxicity occurred.

## 3 Measurements

### 3.1 Efficacy evaluation

CT or MRI was performed after every 1 to 3 chemotherapy cycles based on the evaluation criteria for solid tumors (RECIST Version 1.1) to evaluate primary tumors and liver metastases. A multidisciplinary assessment was performed to assess tumor changes. Surgical resection was performed if the tumor regressed. If the primary tumor progressed between chemotherapy sessions, surgical treatment was promptly performed. Patients who underwent 2 to 8 chemotherapy cycles to evaluate the progression of hepatic metastases were treated with surgery or continued chemotherapy. Outcome endpoints included complete response (CR), partial response (PR), stable disease, and progressive disease; the sum of CR and PR was the clinical response, and CR + PR + stable disease was the disease control rate.

### 3.2 Statistical analysis

All statistical analyses were performed using IBM SPSS 20.0. Data were summarized using the mean for quantitative variables, and counts and percentages for qualitative variables. Continuous variables were tested using *t* tests with independent samples, and categorical variables using the Chi-square test or Fisher exact test. Statistical significance was set at *P* < .05.

## 4 Results

### 4.1 Comparison of general characteristics between neoadjuvant systemic and intra-arterial chemotherapy groups

The clinical and pathological characteristics of the 2 patient groups are shown in Table 1. There were 62 patients (female: 24, male: 38) in intravenous chemotherapy (IVC) group with the mean age of 54.3 ± 9.4.

**Table 1:** Clinical and demographic patient characteristics.

Patient characteristics		IVC group	Pct. (%)	IAC group	Pct. (%)	*P*-value
Number (N)		62		21		
Sex						
	Male	38	61.29	12	57.14	.866
	Female	24	38.71	9	42.86	.827
Age (mean ± SD)		54.3 ± 9.4		48.2 ± 13.7		
	>60	18	29.03	8	38.10	.582
	35–60	44	70.97	13	61.90	.735
Primary tumor location						
	Cecum/ascending colon	9	14.52	0	0.00	.086
	Transverse colon	5	8.06	0	0.00	.197
	Descending colon	7	11.29	2	9.52	.839
	Sigmoid colon	28	45.16	8	38.10	.719
	Rectum	13	20.97	11	52.38	.053
T stage						
	T1–2	12	19.35	6	28.57	.485
	T3–4	50	80.65	15	71.43	.754
N stage						
	N0	8	12.90	5	23.81	.235
	N1	49	79.03	15	71.43	.794
	N2	5	8.06	1	4.76	.849
Pathology results						
Adenocarcinoma						
	Highly differentiated	9	14.52	2	9.52	.560
	Medium differentiation	31	50.00	11	52.38	.850
	Poor differentiation	17	8.06	5	23.81	.756
	Mucinous adenocarcinoma	5	8.06	3	14.29	.404

IAC = intraarterial chemotherapy, IVC = intravenous chemotherapy, Pct. = percentage.

Among them, 14.52% patients (n = 9) occurred in cecum or ascending colon, 8.06% (n = 5) occurred in transverse colon, 11.29% (n = 7) occurred in descending colon, 45.16% (n = 28) occurred in sigmoid colon, and 20.97% (n = 13) occurred in rectum. At the same time, there were 21 patients (female: 9, male: 12) in intraarterial chemotherapy (IAC) group with the mean aged of 48.2 ± 13.7. The primary tumor location of 52.38% (n = 11) occurred in rectum, 38.10% (n = 8) occurred in sigmoid colon, and 9.52% (n = 2) occurred in descending colon. There were no significant differences in sex, age, primary tumor location. 80.65% (n = 50) of patients in IVC group and 71.43% (n = 15) of patients in IAC group were in T3 to T4 stage. 79.03% (n = 49) of patients in IVC group and 71.43 (n = 15) of patients in IAC group were in N1 stage. Both groups were pathologically diagnosed as medium adenocarcinoma mostly (50.00% in IVC group and 52.38% in IAC group). There were no significant differences in T stage, N stage, histological type, etc between groups.

### 4.2 Comparison of efficacy between neoadjuvant systemic and intra-arterial chemotherapy groups

**Figure 1:**
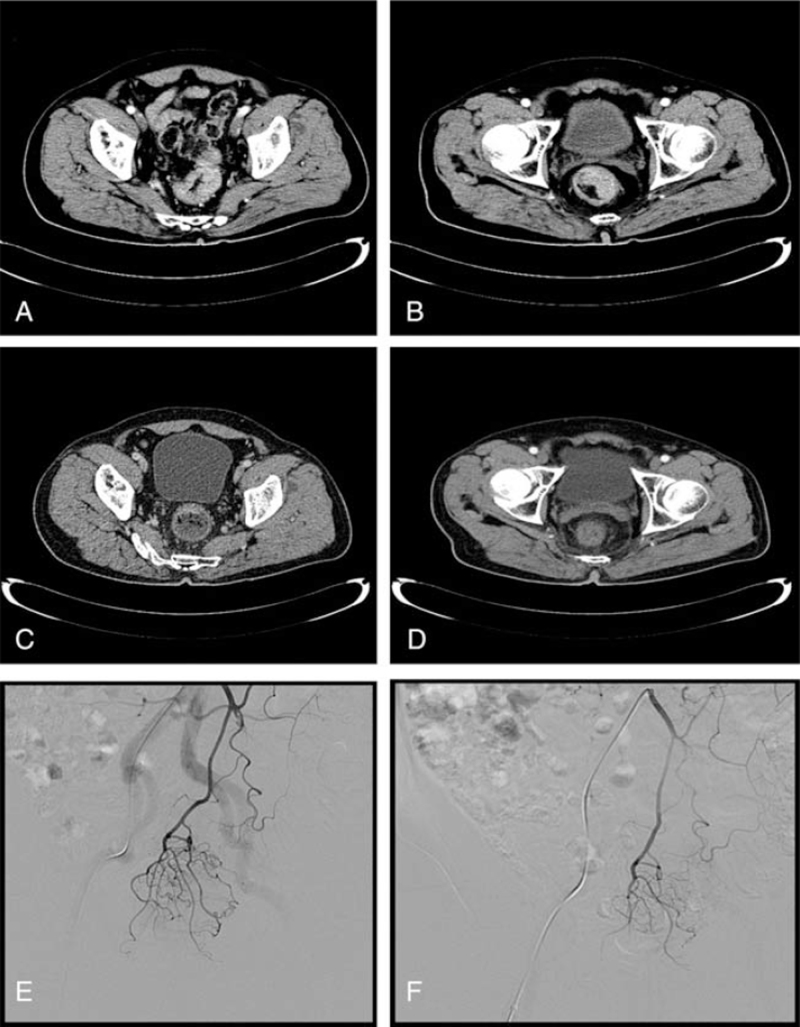
Colorectal cancer. Selected axial images of a contrast-enhanced (CT) scan of the abdomen demonstrate isodense mass on the rectum at (A, B**)** baseline (before chemotherapy) and (C, D**)** follow-up after chemotherapy. A digital subtraction arteriogram via IMA, contrast injection reveals thick, highly tortuous tumor vasculature and obvious contrast staining on the tumor (E) (first chemotherapy) compared to (F) follow- up second chemotherapy.

**Figure 2:**
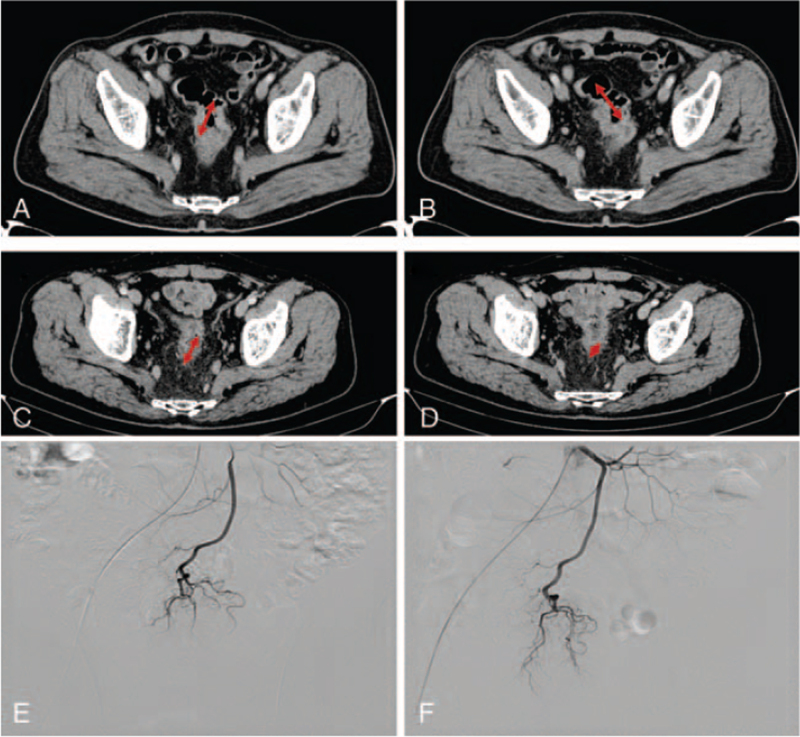
Colorectal cancer. Selected axial images of a contrast-enhanced (CT) scan of the abdomen demonstrate an isodense mass on the colon at (A, B**)** baseline (before chemotherapy) and (C, D**)** follow-up after chemotherapy. A digital subtraction arteriogram via IMA, contrast injection reveals a thick, highly tortuous tumor vasculature and obvious contrast staining on the tumor (E) (first chemotherapy) compared to (F) follow-up to second chemotherapy. This lesion decreased in size in its long-axis diameter resulting in a partial response.

**Figure 3:**
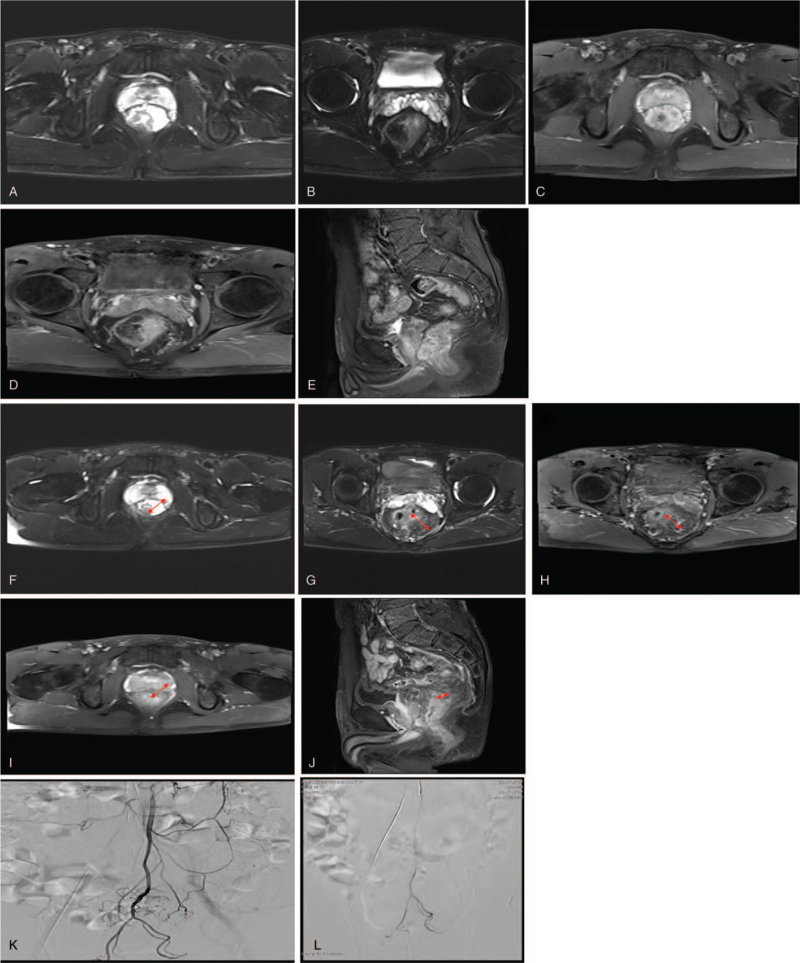
Colorectal cancer. Selected axial (A–D) and sagittal (E) MRI images demonstrate a mass on the colon at (A–E) baseline (before chemotherapy) and (F–J) follow-up after chemotherapy. A digital subtraction arteriogram via IMA, contrast injection reveals a thick, highly tortuous tumor vasculature and obvious contrast staining on the tumor (K) (first chemotherapy) compared to (L) follow-up second chemotherapy. This colorectal carcinoma decreased its size in its long-axis diameter resulting in a partial response.

**Figure 4:**
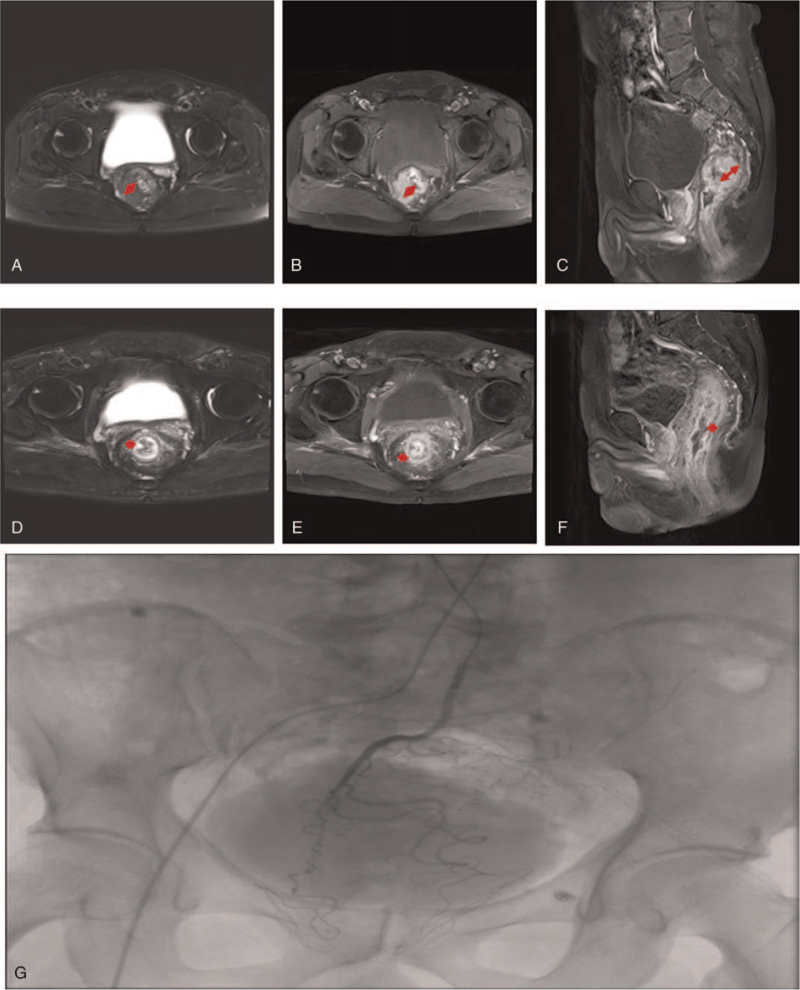
Colorectal cancer. Selected axial (A, B) and sagittal (C) MRI images demonstrate mass on colon at (A–C) baseline (before chemotherapy) and (D–F) follow-up after chemotherapy. A digital subtraction arteriogram via IMA, contrast injection reveals a thick, tortuous tumor vasculature and obvious contrast staining on the tumor (G). This colorectal carcinoma decreased its size in its long-axis diameter resulting in a partial response.

**Figure 5:**
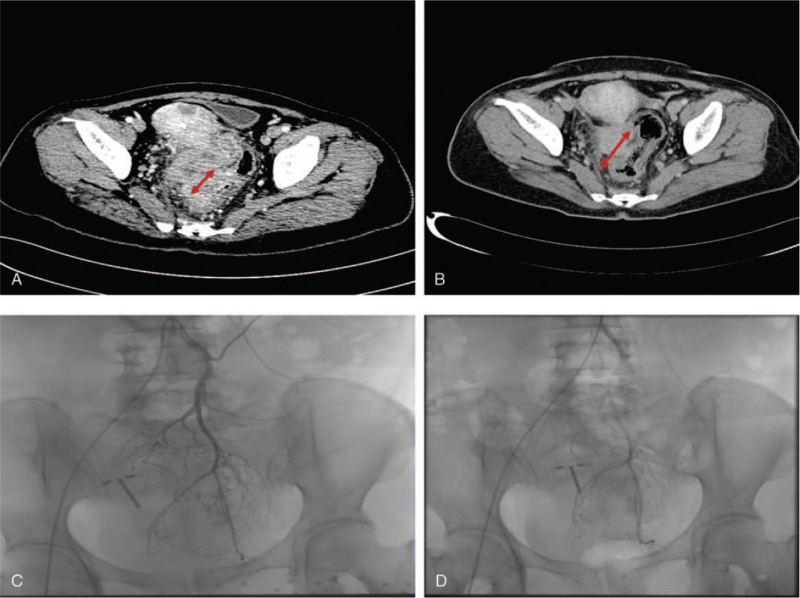
Colorectal cancer. Selected axial images of a contrast-enhanced (CT) scan of the abdomen demonstrate isodense mass on sigmoid colon at (A) baseline (before chemotherapy) and (B) follow-up after chemotherapy. A digital subtraction arteriogram via IMA, contrast injection reveals a thick, highly tortuous tumor vasculature and obvious contrast staining on the tumor (C) (first chemotherapy) compared to (D) follow-up second chemotherapy. This colorectal carcinoma decreased its size in its long-axis diameter resulting in a partial response.

Clinical response was 51.61% in the intravenous chemotherapy group and 71.43% in the arterial chemotherapy group (*P* = .113). The clinical control rate was 80.65% in the intravenous chemotherapy group and 90.48% in the arterial chemotherapy group. The differences between the 2 groups were statistically significant. These results are shown in Table 2 and Figures 1–5.

**Table 2:** Lesion measurement (tumor response) with RECIST evaluation.

RECIST	IVC group	IAC group
CT: tumor maximum diameter before chemotherapy (cm)	3.52 ± 0.69	3.44 ± 1.11
CT: tumor thickest diameter (cm)	2.21 ± 0.43	2.12 ± 0.82
CT: tumor maximum diameter post chemotherapy (cm)	2 .45 ± 0.59	1.87 ± 0.91
CT: tumor thickest diameter (cm)	1.85 ± 0.57	1.19 ± 0.43

RECIST evaluation	IVC group	Pct. (%)	IAC group	Pct. (%)
CR	0	0.00	0	0.00
PR	32	51.61	15	71.43
SD	18	29.03	4	19.05
PD	12	19.35	2	9.52

CR = complete response, CT = computed tomography, IAC = intraarterial chemotherapy, IVC = intravenous chemotherapy, Pct. = percentage, PD = progressive disease, PR = partial response, SD = stable disease.

### 4.3 Treatment toxicity

No patient in this study tolerated chemotherapy due to its associated adverse effects (Tables 3 and 4).

**Table 3:** Changes of hematology parameters on 1^st^ week, 3^rd^ week, and after 3^rd^ week of NACT.

Hematological toxicity	IVC group	Pct. (%)	IAC group	Pct. (%)
Decrease in WBC (1 wk on CT × <3.5 × 10^9^)	9	14.52	0	0.00
Decrease in WBC (3 wk on CT × <3.5 × 10^9^)	14	22.58	2	9.52
Decrease in PLT (1 wk on CT × (125–350 × 10^9^)	3	4.58	1	4.76
Decrease in PLT (3 wk on CT×)	8	12.90	0	0.00
Anemia (3 wk post CT×)	2	3.23	0	0.00
Nonhematological toxicity (1 wk of CT×)				
Loss of appetite	44	70.97	7	33.33
Nausea	49	79.03	7	33.33
Vomiting	32	51.61	2	9.52
Diarrhea	13	20.97	1	4.76
Abnormal LFTs	7	11.29	1	4.76
Peripheral neurotoxicity	6	9.68	1	4.76
Rash	4	6.45	0	0.00
Infection	0	0.00	0	0.00

CT = computed tomography, IAC = intraarterial chemotherapy, IVC = intravenous chemotherapy, LFT = liver function tests, NACT = neoadjuvant chemotherapy, Pct. = percentage, PLT = blood platelet.

**Table 4:** Surgical complication parameters between 2 groups.

Surgical complication	IVC group	Pct. (%)	IAC group	Pct. (%)
Abdominal infection	7	11.29	3	14.29
Anastomotic fistula	4	6.45	1	4.76
Anastomotic bleeding	1	1.61	0	0.00
Intestinal obstruction	4	6.45	1	4.76

IAC = intraarterial chemotherapy, IVC = intravenous chemotherapy, Pct. = percentage.

## 5 Discussion

With increasing incidence and mortality, CRC is the third most common leading cause of death in Western countries and the fifth in China; therefore, it is a major public health problem.^[8]^ Continuous improvements in new adjuvant chemotherapy modalities have improved the quality of life of patients with unresectable CRC and even enabled conversion to resectability. Recently, image-guided transcatheter arterial chemotherapy has received great attention as a local therapy for anti-cancer drug administration.^[5,7]^ Herein, we compared RAIC and intravenous chemotherapy with TOMOX in the treatment of patients with CRC.

Intravenous chemotherapy for CRC is less effective. When the anticancer drug reaches the target lesion after a long route since intravenous injection, a considerable drug amount binds to plasma proteins in the blood and decreases the amount of biologically active free drug. The concentration of the drug in the lesion area is not sufficient to effectively kill the residual cancer cells, resulting in a decrease in intravenous chemotherapy efficacy which may lead to relapse and metastasis in the short term. However, by the transcatheter arterial infusion method, anticancer agents are directly injected through the target vessel, where they are conveniently able to reach the target organ and the drug–protein binding rate is much lower than with intravenous administration. The drug titer can be increased by 2 to 20 times and the curative effect increased by 4 to 10 times.^[9]^ The efficacy of tumor chemotherapy is proportional to the drug concentration. In our study, the local drug concentration by transcatheter arterial infusion was obviously higher than with intravenous chemotherapy, and its efficacy also improved significantly. In this study, the clinical efficacy rate in the arterial infusion chemotherapy group and the intravenous chemotherapy group was similar, indicating that the arterial infusion chemotherapy group did not have a significant advantage over the intravenous chemotherapy group. However, the clinical control rate was significantly better in the arterial infusion chemotherapy group than in the intravenous chemotherapy group (*P* < .05), indicating that the efficiency and tumor control in the first group was higher than in the second.

Transcatheter arterial infusion chemotherapy not only increases the drug concentration in the lesion area and relatively prolongs the duration of the drug action, but also maximizes the drug impact on the lesion. In addition, it decreases the peripheral blood drug concentration, thereby reducing adverse drug reactions. A comparison was performed by Liu et al^[10]^ where 60 cases of colorectal liver metastases were assigned to either receive arterial infusion chemotherapy or systemic chemotherapy. The results showed that the clinical efficacy and clinical benefit rate of the hepatic arterial chemoembolization group were significantly higher than those of the systemic chemotherapy group (*P* < .05), the incidence of adverse reactions significantly lower, and the survival rate at 1, 2, and 3 years and the median survival time higher than in the systemic intravenous chemotherapy group (*P* < .05). In this study, the incidence of adverse effects of arterial infusion chemotherapy was significantly lower than in the intravenous chemotherapy group (*P* < .05). However, the incidence of postoperative complications in the intravenous chemotherapy group was 25.8%, slightly higher – but not significantly so – than that in the arterial infusion chemotherapy group (23.81%).

The limitation of the present study is that it was a single-center retrospective study with a small sample size and short follow-up time for clinical outcomes. However, it was the first to compare the feasibility, efficiency, and toxicity of arterial chemotherapy and systemic chemotherapy with TOMOX in CRC patients, and the present results are useful as they will provide new directions for clinical practice.

## 6 Conclusion

Preoperative interventional arterial infusion chemotherapy in patients with CRC is safe, feasible, and more effective than conventional preoperative systemic chemotherapy in improving the quality of life, as well as improving clinical control while reducing adverse reactions and complications.

## Author contributions

**Conceptualization:** En-Hua Xiao.

**Data curation:** En-Hua Xiao.

**Investigation:** Yan-Hui Li, Zhu Chen.

**Methodology:** Hussein Said Mbarak.

**Resources:** Cong Ma.

**Software:** Shu hui Peng, Quan-Liang Shang, Du-Jun Bian.

**Supervision:** Quan-Liang Shang, Du-Jun Bian.

**Validation:** Zhu Chen.

**Writing – original draft:** Hussein Said Mbarak, Yan-Hui Li.

**Writing – review & editing:** Shu hui Peng, Hussein Said Mbarak.
